# Longitudinal Assessment of Motor Recovery of Contralateral Hand after Basal Ganglia Infarction Using Functional Magnetic Resonance Imaging

**DOI:** 10.1155/2016/7403795

**Published:** 2016-03-16

**Authors:** Yue Fu, Quan Zhang, Chunshui Yu, Jing Zhang, Ning Wang, Shanhuai Zuo, Ningnannan Zhang

**Affiliations:** Department of Radiology, Tianjin Medical University General Hospital, Tianjin 300052, China

## Abstract

We used functional fMRI to study the brain activation during active finger movements at different time points during the recovery phase following basal ganglia infarction. Four hemiplegic patients with basal ganglia infarction were serially evaluated at different time points spanning the acute and chronic phase using fMRI. To evaluate motor recovery, the patients were asked to perform functional tasks arranged in a block design manner with their hand. On follow-up (chronic phase), three patients achieved significant recovery of motor function of affected limbs. Activation of bilateral sensorimotor cortex (SMC) was observed in two of these patients, while activation of cerebellum was observed in all patients. No remarkable recovery of motor function was noted in one patient with left basal ganglia infarction. In this patient, the activation domain was located in SMC of both sides in acute phase and in ipsilateral SMC in chronic phase. Contralateral SMC appears to be involved in the functional rehabilitation following basal ganglia infarction. The cerebellum may act as an intermediary during functional recovery following basal ganglia infarction. The activation domain associated with active finger movement may be bilateral in acute phase; one patient was ipsilateral in the chronic stage.

## 1. Introduction

Motor disturbances are common in patients with cerebral infarction, and motor deficiency in hands results in significant functional disability. More than 75% of patients with cerebral infarction have motor impairment. Approximately 10% of all patients with cerebral infarction end up with a disability due to severe motor impairment [1]. Basal ganglia have neuronal connections with the cerebral cortex, thalamus, brainstem, and several other areas of the brain. The basal ganglia control voluntary motor activity primarily by regulating the motor and premotor cortex [2]. Although the occurrence of motor disturbance following basal ganglia infarction is well recognized, the rehabilitation of motor function has not been serially assessed with longitudinal fMRI [3–5]. This assessment can help in understanding the mechanism of functional recovery, in addition to providing potentially useful metrics for monitoring the efficacy of rehabilitation therapy in these patients.

Blood-oxygen-level dependent-functional magnetic resonance imaging (BOLD-fMRI) is a noninvasive technique that can measure hemodynamic response (change in blood oxygenation and blood flow) related to neural activity in the brain. BOLD-fMRI has been widely used to investigate functional rehabilitation following brain injury [6–8].

In this longitudinal study, we used fMRI to investigate brain activation in relation to voluntary finger movements following basal ganglia infarction. The serial functional imaging data of individual patients that were recorded over a long period of time were analyzed.

## 2. Materials and Methods

### 2.1. Patients

This study was approved by the Institutional Review Board and Ethics Committee of our hospital. Four patients with hemiplegia were enrolled in the study (three males and one female; age range, 59–65 years). These patients were diagnosed with basal ganglia infarction and underwent treatment at the Department of Neurology in our institution between January 2012 and June 2014. Informed consent was obtained from all patients and/or their relatives.

Inclusion criteria were (1) newly diagnosed hemiplegic patients with basal ganglia infarction, (2) absence of other psychological and neurological disorders, and (3) right-handed patients evaluated according to the Chinese handedness criteria. Patients with impaired consciousness, aphasia, and ambidextrous were excluded from this study, such as musicians or keyboard players. According to clinical staging criteria, the clinical course of the illness was classified into three phases: acute phase (≤3 days), subacute phase (4–10 days), and chronic phase (≥11 days) [9]. No drugs were administered to the patients within five hours prior to the examination. Muscle strength in the affected hands was assessed according to the Fugl-Meyer scoring system [10].

### 2.2. Movement Task

Functional tasks (voluntary finger opposition movements with the affected hand) were arranged in a block design. Patients were asked to attempt to use their thumbs in an effort to touch the four fingers repeatedly. If patients with severe motor disability (muscle strength = grade 0; Case  1 in acute phase, Case  2 in acute phase, and Case  3 in chronic phase) were incapable of performing voluntary finger opposition movement, they were asked to imagine the finger opposition movement of the affected hand [11]. Each task consisted of six blocks of 20 seconds including three rest blocks and three movement blocks. The tasks started with the rest block, followed by alternating movement and rest blocks. During the rest blocks, patients were asked to remain motionless with both hands resting by the side of the body with quiet breathing. During the movement blocks, patients were asked to perform the finger opposition movement twice in one second (0.5 Hz), with the wrist and arm held still. For Cases  1–3, fMRI examinations were performed in the acute phase and in the chronic phase, respectively. For Case  4, the patient underwent one fMRI examination in the acute phase and two fMRI examinations in the chronic phase.

### 2.3. Radiological Examinations and BOLD-fMRI

MRI was performed on a GE 1.5-T MRI system (GE 1.5T Twin-speed infinity with Excite II scanner, GE, USA). Anatomical images of the whole head were acquired with a T1-weighted gradient echo pulse sequence. Axial T1-weighted imaging was conducted using a fast fluid-attenuated inversion recovery (FLAIR) sequence. The following parameters were used: repetition time (TR), 2732.9 ms; echo time (TE), 11.6 ms; inversion time (IT), 760 ms; bandwidth (BH), 19.23 KHz; field of view (FOV), 24 × 18 cm; matrix, 320 × 224; slice thickness, 6 mm; interslice gap, 1 mm; 20 slices covering the whole brain.

Routine MRI and diffusion weighted imaging (DWI) were performed to evaluate radiological characteristics of the basal ganglia infarction. Parameters for DWI included the following: TR/TE, 10,000/64 ms; FOV, 256 × 256 mm; matrix, 128 × 128; slice thickness, 3 mm; interslice gap, 0 mm; *b* value, 1,000 s/mm^2^; 45 slices covering the whole brain. Furthermore, diffusion tensor imaging (DTI) was performed on Case  4 to analyze tractography changes. DTI was acquired with a single-shot spin-echo echo-planar imaging (SS-SE-EPI) sequence (TR/TE, 10,000/64 ms; FOV, 256 × 256 mm; matrix, 128 × 128; slice thickness, 3 mm; interslice gap, 0 mm; *b* value, 1,000 s/mm^2^; 45 slices covering the whole brain).

Functional MRI was performed using gradient echo (GRE) and a single-shot echo-planar imaging (EPI) sequence. Parameters were as follows: TR, 3,000 ms; TE, 40 ms; inversion angle, 90°; BH, 62.50 KHz; FOV, 24 × 24 cm; matrix, 128 × 96; intraslice resolution, 1.875 × 2.5 mm; slice thickness, 6 mm; interslice gap, 1 mm; 20 slices covering the whole brain matching the T1-weighted slices.

### 2.4. Data Analysis

Preprocessing and statistical analyses of fMRI data were performed with SPM5 (statistical parametric mapping, Wellcome Department of Cognitive Neurology, University College, London, UK). Data preprocessing parameters included motion correction and spatial smoothing. Data from each patient were independently analyzed without resorting to standardization. After preprocessing, *t*-tests across pixels were performed to obtain statistical parametric maps. *P* < 0.05 was considered statistically significant. The threshold of activated pixels was set as ≥5 pixels, indicating that regions with ≥5 continuously activated pixels were effectively considered as activated brain areas. Localization of activated pixels in specific brain regions was achieved using T1-weighted images, and BOLD-fMRI data were registered to the subject's anatomical structures. The *t*-values from statistical analyses were used to reflect activation intensity, and the activation range was calculated by the summation of the number of pixels in each activated brain area. DTI data was analyzed with the FMRIB Software Library (FSL, FMRIB, University of Oxford, Oxford, UK).

## 3. Results

### 3.1. Longitudinal Observation in Patients with Motor Functional Recovery

In Cases  1–3, basal ganglia infarction was localized in the unilateral hemisphere, and the infarction-contralateral limb was affected. In Case  4, ischemic appearance was observed in the bilateral basal ganglia on MRI. The left basal ganglia was diagnosed as infarction and the right limb was affected, while DWI revealed spot-like hyperintensity in the right basal ganglia, suggesting small ischemic areas; and the left limb was not affected.

During the follow-up period (chronic phase), motor function of the affected (infarction-contralateral) limb was significantly recovered in three patients. Repeated fMRI examinations in the chronic phase revealed the activation of the bilateral sensorimotor cortex (SMC) in two patients, which indicate the expansion of the activation range, compared to unilateral activation in the acute phase. Activation of the cerebellum was observed in all three patients. Data is summarized in Table 1.

#### 3.1.1. Patient 1

**Figure 1:**
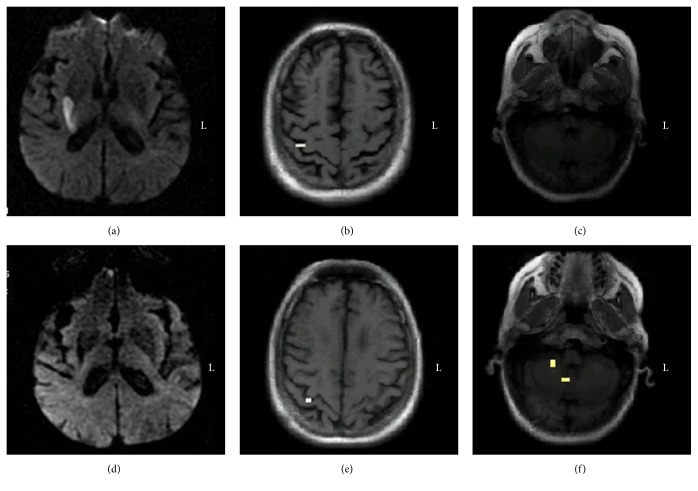
The longitudinal study of brain activation in Case 1 during the voluntary finger opposition task. (a) Diffusion weighted images on the first day of onset showing infarction of the right basal ganglia; (b and c) fMRI in the acute phase demonstrating the activation of the right SMC; (d) diffusion weighted images seven months after onset showing the encephalomalacia foci in the right basal ganglia; (e and f) serial fMRI in the chronic phase demonstrating the activation of the right SMC and right cerebellum. fMRI, functional magnetic resonance imaging; SMC, sensorimotor cortex.

This patient had right basal ganglia infarction. On the first day of onset, muscle strength was grade 0, and fMRI revealed right SMC activation. After seven months, muscle strength recovered to grade 1, and repeat fMRI revealed the activation of the right SMC and right half of the cerebellum (Figure 1).

#### 3.1.2. Patient 2

**Figure 2:**
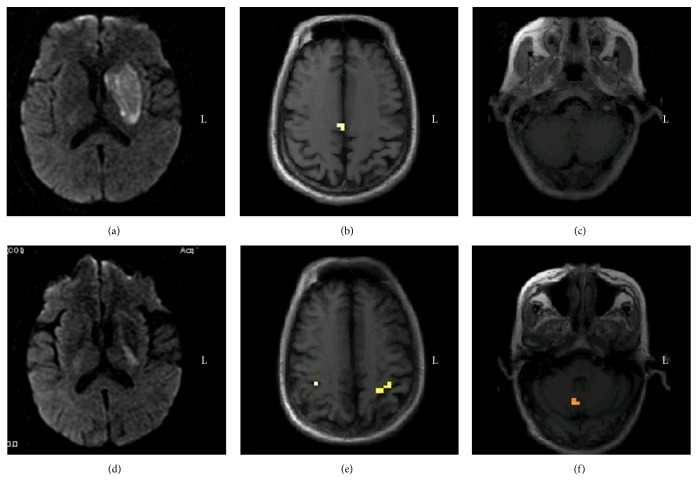
Longitudinal observation of brain activation in Case  2 during the voluntary finger opposition task. (a) Diffusion weighted images on the third day after onset showing infarction of the left basal ganglia; (b and c) fMRI in the acute phase demonstrating the activation of the bilateral SMA; (d) diffusion weighted images two months after onset showing the encephalomalacic foci in the left basal ganglia; (e and f) serial functional MRI in the chronic phase demonstrating the activation of the bilateral SMC and right cerebellum. fMRI, functional magnetic resonance imaging; SMA, supplementary motor area.

This patient had left basal ganglia infarction. On the third day after onset, muscle strength was grade 0, and fMRI revealed bilateral SMC activation. After two months, muscle strength recovered to grade 1, with repeat fMRI showing the activation of SMC on both sides and in right half of the cerebellum (Figure 2).

#### 3.1.3. Patient 3

**Figure 3:**
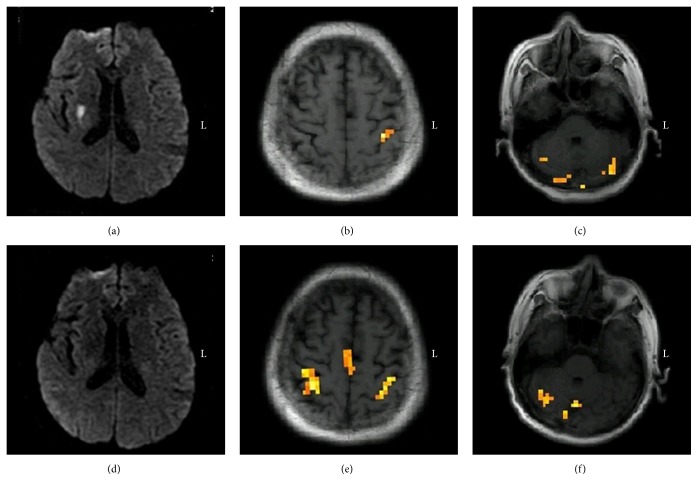
Longitudinal observation of brain activation in Case  3 during the voluntary finger opposition task. (a) Diffusion weighted images on the third day after onset showing infarction of the right basal ganglia; (b and c) fMRI in the acute phase demonstrating the activation of the left SMC and bilateral cerebellum; (d) diffusion weighted images three months after onset showing the encephalomalacia foci in the right basal ganglia; (e and f) serial fMRI in the chronic phase demonstrating the activation of the bilateral SMC, bilateral SMA, and right cerebellum. fMRI, functional magnetic resonance imaging; SMC, sensorimotor cortex; SMA, supplementary motor area.

This patient had right basal ganglia infarction. On the third day after onset, muscle strength was grade 2, and activation of the left SMC and bilateral cerebellum on voluntary movement of the affected fingers was observed. After three months, muscle strength recovered to grade 4, and repeat fMRI examination revealed the activation of the bilateral SMC, right cerebellum, and bilateral supplementary motor area (SMA) (Figure 3).

### 3.2. Longitudinal Observation in the Patient without Significant Motor Functional Recovery

**Figure 4:**
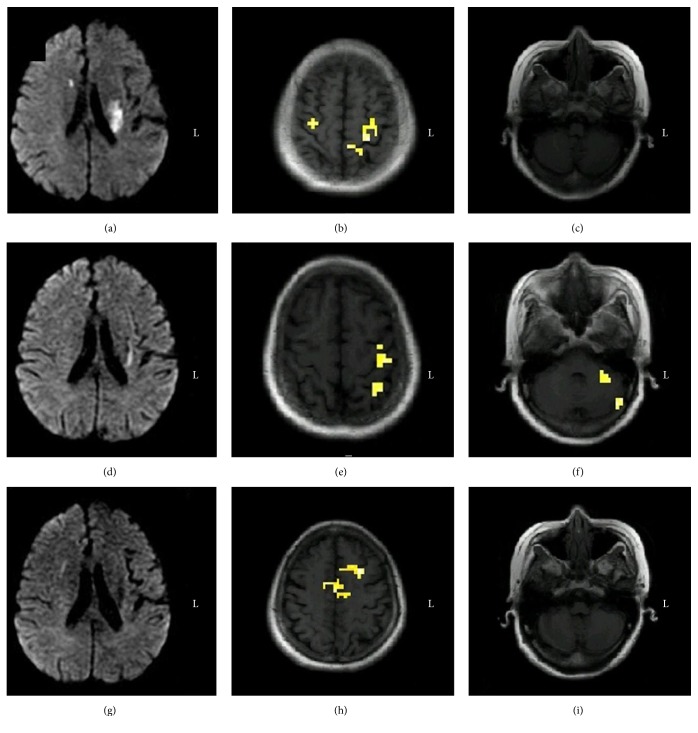
Longitudinal observation of brain activation in Case  4 during the voluntary finger opposition task. (a) Diffusion weighted images on the first day of onset showing infarction of the left basal ganglia; (b and c) fMRI in the acute phase demonstrating the activation of the bilateral SMC; (d) diffusion weighted images three months after onset showing the encephalomalacic foci in the left basal ganglia; (e and f) serial fMRI three months after onset demonstrating the activation of the left SMC, left PPC, and left cerebellum; (g) diffusion weighted images 12 months after onset showing no significant changes of the encephalomalacia foci; (h and i) the third-time fMRI twelve months after onset demonstrating the activation of the bilateral SMA and left PFC. fMRI, functional magnetic resonance imaging; SMC, sensorimotor cortex; SMA, supplementary motor area; PPC, posterior parietal cortex; PFC, prefrontal cortex.

**Figure 5:**
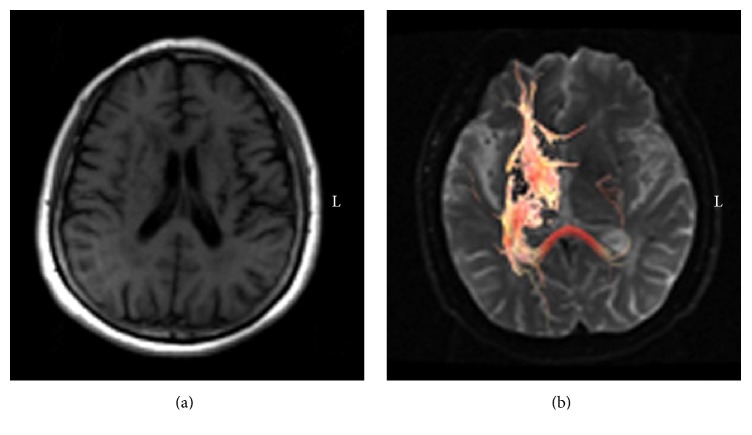
Follow-up radiographs of Case  4 in the chronic phase. (a) T1-weighted images show the encephalomalacic foci in the posterior limb of the left internal capsule; (b) an additional diffusion tensor tractography shows the complete interruption of the corticospinal tract in the posterior limb of left internal capsule.

One patient with left-sided basal ganglia infarction revealed no significant motor functional recovery. On the day of onset, muscle strength was grade 1 and fMRI revealed bilateral SMC activation. After three months, no change in muscle strength was observed; but a repeat fMRI examination revealed the activation of the left SMC, left posterior parietal cortex (PPC), and left cerebellum. Twelve months after onset, muscle strength deteriorated to grade 0, and fMRI revealed the activation of the bilateral SMC and left prefrontal cortex (PFC). Comparison of serial data revealed a reduction in both the intensity and range of activation in the infarction-ipsilateral SMC, and this activation was invisible in the chronic phase (Figure 4). An additional diffusion tensor tractography revealed fibrous interruption of the corticospinal tract in the left posterior limb of the internal capsule (Figure 5).

## 4. Discussion

Movement disorders are common sequelae of basal ganglia infarction that frequently involve hand movements. Longitudinal studies evaluating the temporal evolution of neural recovery using fMRI following basal ganglia infarction have been exceedingly rare. In the present study, we conducted a longitudinal assessment of functional rehabilitation through the acute and chronic phases after basal ganglia infarction. All four patients in this study were diagnosed with basal ganglia infarction without other functional domain involvement. This facilitated the monitoring of dynamic functional rehabilitation without any distractions.

Most previous studies have confirmed the involvement of changes in the cortical functional domain during motor function recovery. However, there is no consensus on the correlation between cortical activation and motor functional rehabilitation, the activated site of the brain, activation intensity, the recovery process, and efficiency of rehabilitation therapy. In this study, we documented the activation of the bilateral SMC in two patients with significant motor functional rehabilitation. Moreover, longitudinal functional MRI examination was able to delineate the expansion of the activation range. The activation of the unaffected SMC during voluntary movements of the affected hands suggests that the SMC of the unaffected hemisphere might have an important role in motor functional recovery following basal ganglia infarction. Jang et al. conducted an fMRI study on a patient with cortical infarction involving the right primary SMC and severe sensorimotor impairment, which completely recovered following rehabilitation therapy at six months from onset. BOLD-fMRI confirmed functional reorganization in the lateral hemisphere. Furthermore, they speculated that the SMA, PPC, and cerebellum could have a mediating effect during functional recovery following cortical infarction [12, 13]. Song et al. reported ipsilateral hemiparesis caused by a left-sided corona radiata infarct in two patients who previously had left-sided hemiparesis due to a contralateral supratentorial infarct. On fMRI, bilateral SMC activation during movement of the paretic left hand was observable. According to the authors, the new onset left-sided hemiparesis was caused by a new infarct in the ipsilateral motor area, which was functionally reorganized after the previous stroke [14].

Animal experiments have demonstrated that task-specific rehabilitative therapy is effective in improving motor function in a rat model of focal ischemia. In one such study, the enhancement in dendritic complexity and length in the contralateral hemisphere was discernible following the institution of rehabilitative therapy, suggesting that the plasticity of the contralateral hemisphere was associated with functional recovery [15]. Shimizu et al. employed transcranial magnetic stimulation in patients with cortical stroke and observed the stimulation of the ipsilateral motor cortex in the early stages of unilateral cortical stroke, which may be caused by the disruption of transcallosal inhibition [16]. Furthermore, the involvement of the contralateral hemisphere in functional rehabilitation was a constant finding. Animal fMRI experiments have revealed that early functional recovery after stroke is related to the unmasking of existing neuronal circuitry or stimulation of the contralesional hemisphere. This is because the formation of new anatomic connections requires several days and is a process that peaks several weeks after stroke [17]. In our study, there was significant activation of the cerebellum in all three patients with recovered motor function in the affected limbs, which is consistent with the findings reported by Small et al. [18]. In their study, Small et al. employed fMRI for studying a case series for longitudinal assessment of motor recovery in patients with stroke. The task they adopted involved finger and wrist movement at four time points during the first six-month stroke recovery phase. Patients with good recovery had observable changes in the activation of the cerebellar hemisphere contralateral to the injured corticospinal tract, while patients with poor recovery did not show any changes. This suggests the role of the cerebellum in mediating poststroke functional recovery.

In the present study, longitudinal fMRI examination in the patient without motor function recovery revealed the activation of the SMC on both sides in the early stages, and the ipsilateral SMC, PPC, and cerebellum were activated at three months after onset. The activation domain of the bilateral SMC was eventually confined to the ipsilateral SMC in the chronic phase, which is consistent with earlier studies that demonstrated an evolution in SMC activation from the early contralesional site to the late ipsilesional site [19–22]. Some reports also support the role of the PPC and cerebellum in mediating motor functional recovery after infarction [13, 18, 23]. In the subsequent year, when no effective rehabilitation therapy was scheduled, muscle strength deteriorated to grade 0. Upon performing an fMRI examination, the patient was instructed to imagine the finger opposition movement of the affected hand; and bilateral activation of the SMA and ipsilateral activation in the PFC were observed. An additional DTT revealed the complete interruption of the corticospinal tract in the left posterior limb of the internal capsule. Activation of the SMA on both sides and activation of the PFC on the affected side confirmed the plasticity of perilesional domains. This recovery may be due to the functional overlapping region in the sensorimotor network. Furthermore, electrophysiological studies indicate that most of the corticospinal tracts were from the primary motor cortex, and there were direct projections from the PMA and SMA as well. A single cell recording study confirmed the substitution effect of the SMA following cortical injury. It is also known that SMA neurons are activated when a motor task is first learned, but this tends to weaken after extensive overlearning. Moreover, after injury to the cortical area, the SMA tends to become activated again upon repeating the previously learned task [24]. These findings are consistent with fMRI studies, demonstrating the activation of the SMA in patients with cerebral infarction [25, 26].

The main mechanisms involved in functional recovery following cerebral infarction are as follows: (a) reorganization of functional movement pathways, namely, the formation of new neuronal circuitry by neuronal sprouting and/or synaptogenesis; (b) unmasking or strengthening of preexisting pathways through disinhibition and/or potentiation; and (c) backward shift of the primary sensorimotor cortex. Cerebral infarction not only induces perilesional changes but also induces obvious functional and structural changes in remote regions. Weiller et al. measured regional cerebral blood flow in patients with cerebral infarction during voluntary and passive movements, both during flexion and extension of the right elbow, using positron emission tomography [27]. They reported brain activation during both conditions in the lesional area, as well as in the SMA, SMC, PMA, PFC, and cingulate gyrus. In the present study, functional and structural changes were obvious in both the perilesional and remote regions during the recovery of motor function. Multiple functional regions were activated during the performance of the task, including the SMC, cerebellum, PMA, SMA, PPC, and PFC. These results confirm the presence of functional reorganization following basal ganglia infarction. SMC activation on both sides could be a manifestation of the tendency for ipsilesional localization over time [21]. The present study only provides a brief analysis, with the small sample size being a significant limitation. Understanding precise reorganization mechanisms would require further studies involving a larger cohort, with follow-up studies over an extended period of time.

## 5. Conclusion

Contralateral SMC is involved in functional rehabilitation following basal ganglia infarction. Cerebellar activity may act as an intermediary during functional recovery courses. Additionally, the activation domain associated with voluntary finger movement may be bilateral during the acute phase but tends to be ipsilateral during the chronic phase. The specific reorganization mechanisms for functional recovery following basal ganglia infarction require further studies.

## Figures and Tables

**Table 1:** Results of serial BOLD-fMRI associated with active finger movement.

Patient number	Site of infarction	Examination sequence	Duration from onset	Phase	Muscle strength	Fugl-Meyer score	Activated regions	Activation intensity	Activation range
1	Right basal ganglia	1	1 day	Acute	0	12	Right SMC	7.21	5
		2	7 months	Chronic	I	19	Right SMC	8.4	5
							Right cerebellum	7.72	6

2	Left basal ganglia	1	3 days	Acute^*∗*^	0	4	Bilateral SMA	6.45	7
		2	2 months	Chronic	I	16	Left SMC	5.35	14
							Right SMC	4.64	5
							Right cerebellum	5.22	5

3	Right basal ganglia	1	3 days	Acute	II	39	Left SMC	12.41	33
							Left cerebellum	12.79	42
							Right cerebellum	12.14	59
		2	3 months	Chronic	IV	60	Right SMC	9.55	9
							Left SMC	14.3	52
							Right cerebellum	9.88	11
							Bilateral SMA	7.99	13

4	Left basal ganglia	1	1 day	Acute	I	10	Left SMC	9.41	13
							Right SMC	8.74	5
		2	3 months	Chronic	I	8	Left SMC	6.58	12
							Left PPC	6.62	8
							Left cerebellum	6.78	14
		3	12 months	Chronic^*∗*^	0	6	Bilateral SMA	4.85	19
							Left PFC	5.81	14

BOLD-fMRI, blood-oxygen-level dependent-functional magnetic resonance imaging; SMC, sensorimotor cortex; SMA, supplementary motor area; PPC, posterior parietal cortex; PFC, prefrontal cortex.

^*∗*^As the patient with severe motor disability (the muscle strength being grade 0) was incapable of performing voluntary finger opposition movement, they were asked to imagine the finger opposition movement of the affected hand.
